# Supplementary material for: Impact of azithromycin and nitazoxanide on the enteric infections and child growth: Findings from the Early Life Interventions for Childhood Growth and Development in Tanzania (ELICIT) trial
Source: PLoS One. 2023 Dec 21;18(12):e0294110. doi: 10.1371/journal.pone.0294110 (PMC10734999; doi:10.1371/journal.pone.0294110)
Supplement: S1 Table — (DOCX) [file pone.0294110.s001.docx]

**Supplemental Table 1.** Pathogens and gene targets tested by PCR in this study.

|  | Pathogen/target | Gene |
| --- | --- | --- |
| Viruses | Adenovirus 40/41 | Fiber gene |
|  | Astrovirus | Capsid |
|  | Norovirus GI | ORF1-2 |
|  | Norovirus GII | ORF1-2 |
|  | Rotavirus | *NSP3* |
|  | Sapovirus | *RdRp* |
| Bacteria | EAEC* | *aaiC, aatA, aggR* |
|  | Atypical EPEC* | *eae* |
|  | Typical EPEC* | *bfpA* |
|  | ETEC* | *LT, STh* and *STp* |
|  | STEC* | *stx1, stx2* |
|  | *Aeromonas* | Aerolysin |
|  | *Bacteroides fragilis* | *ETBF* |
|  | *Campylobacter jejuni* and *C. coli* | *cadF* |
|  | *Campylobacter* | *cpn60* |
|  | *Clostridium difficile* | *tcdA* and *tcdB* |
|  | *Helicobacter pylori* | *ureC* |
|  | *Plesiomonas shigelloides* | *gyrB* |
|  | *Salmonella* | *ttr* |
|  | *Shigella/*EIEC | *ipaH* |
|  | *Shigella flexneri* | Putative periplasmic protein, O-antigen, Type 3 restriction enzyme |
|  | *Shigella sonnei* | Putative methylase |
|  | *Vibrio cholerae* | *hlyA* |
| Fungi | *Enterocytozoon bieneusi* | *ITS* |
|  | *Encephalitozoon intestinalis* | SSU rRNA |
| Protozoa | *Cryptosporidium* | 18S rRNA |
|  | *Cyclospora* *cayetanensis* | 18S rRNA |
|  | *Cystoisospora belli* | 18S rRNA |
|  | *Entamoeba histolytica* | 18S rRNA |
|  | *Giardia* | 18S rRNA |
| Helminth | *Ancylostoma* *duodenale* | *ITS2* |
|  | *Ascaris* *lumbricoides* | *ITS1* |
|  | *Blastocystis* | 18S rRNA |
|  | *Hymenolepis nana* | 18S rRNA |
|  | *Necator* *americanus* | *ITS2* |
|  | *Schistosoma* | 18S rRNA |
|  | *Strongyloides* *stercoralis* | Dispersed repetitive sequence |
|  | *Trichuris* *trichiura* | 18S rRNA |
| Controls | MS2 | *MS2g1* |
|  | PhHV | *gB* |
| Others** | *Campylobacter upsaliensis* | *atpA* |
|  | Rotavirus | VP7 G1, G2, G3, G4, G8, G9, G12, VP4 P[4], P[6], P[8] |
|  | Pan-Adenovirus | Hexon |
|  | Norovirus GI.1  Norovirus GII.4 | ORF1 |
|  | ETEC colonization factors | CFA/I, CS1, CS2, CS3, CS5, CS6 |
|  | *Mycobacterium tuberculosis* | *IS6110* |
|  | *Cryptosporidium hominis*  *Cryptosporidium parvum* | *LIB13* |
|  | *Giardia lamblia* assemblage A and B | *TPI* |
|  | *Entamoeba* spp*.* | 18S rRNA |
| Antimicrobial resistance genes |  | *mphA* |
|  |  | *ermB* |
|  |  | *NDM* |
|  |  | *KPC* |
|  |  | *CTX-M* |
|  |  | *MCR-1* |
|  |  | *OXA* |
|  |  | *23S Campy 2075A & G* |
|  |  | *Campy gyrA 86T & I C.jejuni* |
|  |  | *Campy gyrA 86T & I C.coli* |
|  |  | *Shigella/E.coli gyrA 87D & NY* |
|  |  | *Shigella/E.coli gyrA 83S & L* |
|  |  | *Shigella/E.coli gyrA 87D & G* |
|  |  | *Shigella/E.coli parC 80S & I* |

* *E. coli* pathotypes were defined as follows: EAEC (*aaiC*, or *aatA*, or both), atypical EPEC (*eae* without *bfpA*, *stx1*, and *stx2*), typical EPEC (*bfpA*), ETEC (STh, STp, or LT), STEC (*eae* without *bfpA* and with *stx1*, *stx2*, or both).

**not tested on all samples
