# Supplementary material for: Impact of azithromycin and nitazoxanide on the enteric infections and child growth: Findings from the Early Life Interventions for Childhood Growth and Development in Tanzania (ELICIT) trial
Source: PLoS One. 2023 Dec 21;18(12):e0294110. doi: 10.1371/journal.pone.0294110 (PMC10734999; doi:10.1371/journal.pone.0294110)

**S Fig 1. Difference in pathogen detection in the nicotinamide versus placebo arms at 6, 6.5, 12, 12.5, and 18 months.** X axis is the absolute reduction in pathogen prevalence between the nicotinamide – placebo. Negative values to the left of 0.0 represent reduction with nicotinamide. 95% confidence intervals are included.


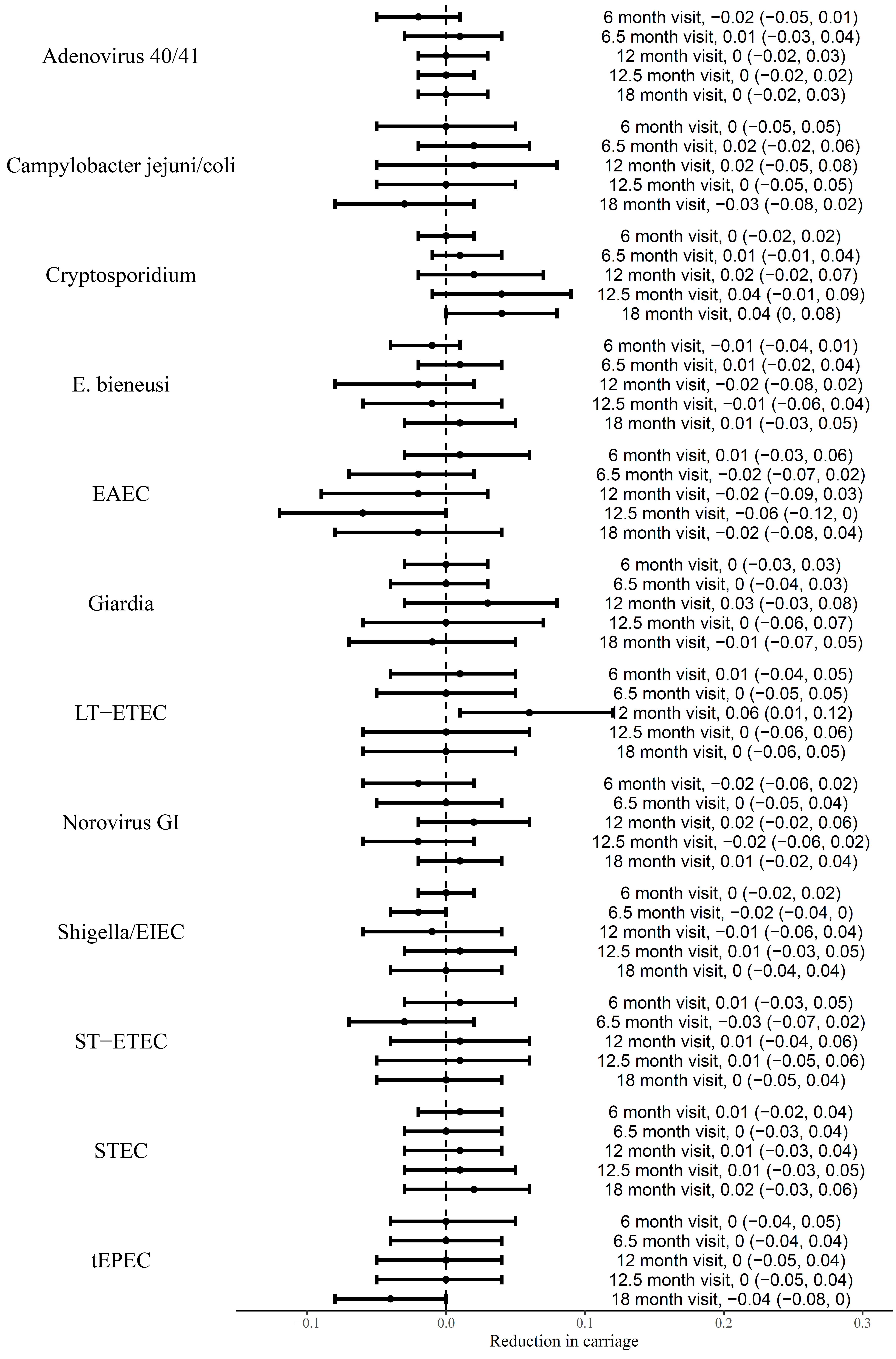

Supplement: S1 Fig — X axis is the absolute reduction in pathogen prevalence between the nicotinamide–placebo. Negative values to the left of 0.0 represent reduction with nicotinamide. 95% confidence intervals are included. (DOCX) [file pone.0294110.s004.docx]
