# Supplementary figures and images for: Impact of azithromycin and nitazoxanide on the enteric infections and child growth: Findings from the Early Life Interventions for Childhood Growth and Development in Tanzania (ELICIT) trial
Source: PLoS One. 2023 Dec 21;18(12):e0294110. doi: 10.1371/journal.pone.0294110 (PMC10734999; doi:10.1371/journal.pone.0294110)

**S Fig 2. Prevalence of non-study antibiotic use by drug class and month.**


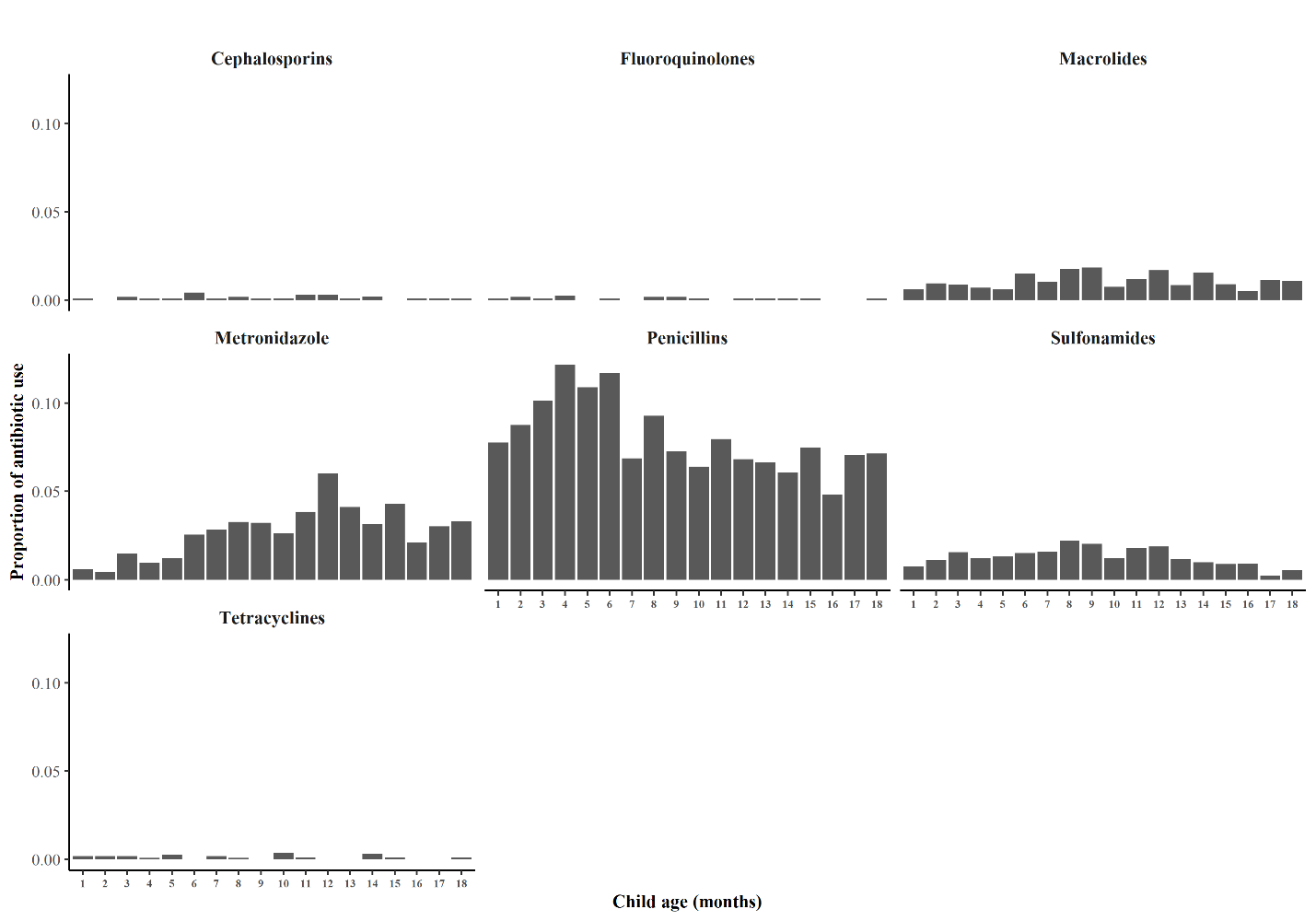

Supplement: S2 Fig — (DOCX) [file pone.0294110.s005.docx]
